# A proposal for a self-rated frailty index and status for patient-oriented research

**DOI:** 10.1186/s13104-019-4206-3

**Published:** 2019-03-25

**Authors:** Yi-Sheng Chao, Danielle McGolrick, Chao-Jung Wu, Hsing-Chien Wu, Wei-Chih Chen

**Affiliations:** 1 Canadian Agency for Drugs and Technologies in Health, Ottawa, Canada; 2 Département d’informatique, Université du Québec à Montréal, Montréal, Canada; 3 Taipei Hospital, Ministry of Health and Welfare, New Taipei City, 242 Taiwan; 4 Department of Chest Medicine, Taipei Veterans General Hospital, Taipei, Taiwan; 5 Faculty of Medicine and Institute of Emergency and Critical Care Medicine, School of Medicine, National Yang-Ming University, Taipei City, Taiwan

**Keywords:** Frailty, Frailty index, Frailty status, Self-rated frailty measure, Phenotype Model, Accumulation of Deficits Model

## Abstract

**Objective:**

Frailty indices are important predictors of major health outcomes, but mostly designed by and for researchers and specialists. Three of the most commonly used theory-based indices are composite measures that are subject to arbitrary assumptions and biases introduced due to data processing. A complicated index can be simplified with fewer items. The theory-based frailty indices are not optimal and neglect patients’ perspectives. This study aims to compare different definitions of frailty and propose a self-rated measure of frailty index and status.

**Results:**

Frailty was defined differently by laypeople and researchers/clinicians. Patients’ and laypeople’s perspectives seemed neglected. Existing frailty indices had shortcomings related to the use of composite measures, assumptions of frailty theories, and the lack of novel information. To avoid these shortcomings, we suggested asking individuals “on a scale of 0 to 10, how frail do you think you are?” and “by answering yes or no, do you consider yourself to be frail?” to determine frailty on continuous and dichotomous scales respectively. However, there will be other issues emerging with these new measures, such as the need for feasibility and validity studies, as well as acceptability by researchers.

## Introduction

Frailty is often considered a geriatric syndrome by researchers [1, 2], characterized by weakness, slow gait, and other aging-related symptoms or diseases [1–3]. In a 2018 review, the mostly commonly adopted theory was the Phenotype Model by Fried et al., followed by the Accumulation of Deficits Model by Rockwood et al. [4]. According to the Phenotype Model, frailty can be defined if at least three of the following present: weight loss, low handgrip strength, slow walking speed, exhaustion, and reduced physical activity [5]. The frailty measured by the Accumulation of Deficits Model is defined with 70 variables that characterize the deficits in cognition, cardiovascular system, metabolism, and others [6, 7]. Despite their popularity in the research field, these two models have been recognized for problems in the assumptions they rely on and the biases introduced by data processing [1].

The assumptions made in the creation of the two frailty indices by the two models include assigning input variables with equal weights, arbitrary age criteria, and the lack of evidence for formulating the thresholds for frailty statuses based on frailty indices [1]. The input variables have been conventionally recognized as equally important and assigned equal weight to derive frailty indices [1]. Recent studies suggest that this assumption needs to be tested [1]. The age criteria for the diagnosis of frailty proposed by the two models differ, a minimum age of 65 and 70 years respectively [1, 2]. Lastly, the proposed thresholds that define frailty statuses and are used to determine the prevalence estimates differ, at 0.6 and 0.2 respectively [1]. These thresholds may also influence the magnitudes of the biases that can be introduced to the indices [1].

Furthermore, these frailty indices defined by the two models are subject to interpretability problems for different reasons [1]. The Phenotype Model relates to biases generated purely by data processing [1]. Bias alone can explain more than 71% of the variance of the frailty index defined by the Phenotype Model [1]. The 70-item frailty index defined by the Accumulation of Deficits Model can be further simplified with fewer input variables [1]. This suggests that many input variables may in fact be similar or highly correlated [1]. As a result, these two models fail to reflect the theories they are based on [1].

In addition, patients’ perspectives are increasingly emphasized [8]. The two frailty models are generated mostly for research purposes using administrative or survey data [2]. Research-oriented frailty indices have been criticized for focusing purely on the clinical aspects of this aging phenomenon [3]. The frailty indices defined by the two models may not be closely linked to patients’ perception. To improve the research on frailty, this study aims to (1) highlight the differences in frailty definitions adopted by laypeople and clinicians/researchers, and (2) propose self-rated frailty measures that avoid the shortcomings existing in many research-oriented indices.

## Main texts

A search for definitions of frailty was performed and the results were listed in Table 1. Frailty could be defined from at least two perspectives: layperson and researcher/clinician. From a layperson’s perspective, frailty was often referred to as weakness, delicacy, and being fragile [9, 10]. For statisticians, frailty models were those that took random effects into consideration and frailty was used to describe the unobserved heterogeneity of a deterioration of health [11]. Since the 1990s, statisticians have used frailty to refer to random effects in statistical models, especially survival analysis [11]. For aging researchers and clinicians, frailty became a popular research topic in medical research since the 2000s; however a variety of frailty definitions have been used [3]. According to an expert consensus, frailty could be defined as “a clinical syndrome characterized by declining reserve and diminished resistance to stressors” [3, 12]. Compared to the layperson’s perspective, which primarily links frailty to physical weakness, researchers and clinicians often consider frailty in terms of aging and interaction with stressors or physical environments according to the three definitions used by frailty researchers [3]. Although the Accumulation of Deficits Model did not explicitly link frailty to aging or interaction with external factors, this model also set up age threshold for the diagnosis of frailty [2, 3].

**Table 1:** Frailty defined by the public, statisticians and aging researchers

Perspectives	Definitions
Laypeople	“The quality or state of being frail” (frail: easily broken or destroyed; physically weak) [9]
	“The condition of being weak and delicate” [10]
Researchers/clinicians	The random effects that “account for association and unobserved heterogeneity” in statistical models [11]
	“A state of vulnerability that becomes more prevalent with age and affects an individual’s resilience and ability to deal with minor and major stressors, which can include illnesses or infections” (National Institute on Ageing definition) [3]
	“A clinical syndrome characterized by declining reserve and diminished resistance to stressors” (expert consensus) [12]
	“A phenotype, which is defined as an individual’s observable traits that result from the interaction of their genetic information with their physical environment.” (Phenotype Model) [3]
	“An accumulation of deficits, which can be physical, cognitive, and clinical challenges an individual may be facing, including falls, changes in the ability to carry out everyday activities, depression, restlessness, memory changes, and congestive heart failure—the more deficits an individual has, the greater their level of frailty” (Accumulation of Deficits Model) [3]

**Fig. 1:**
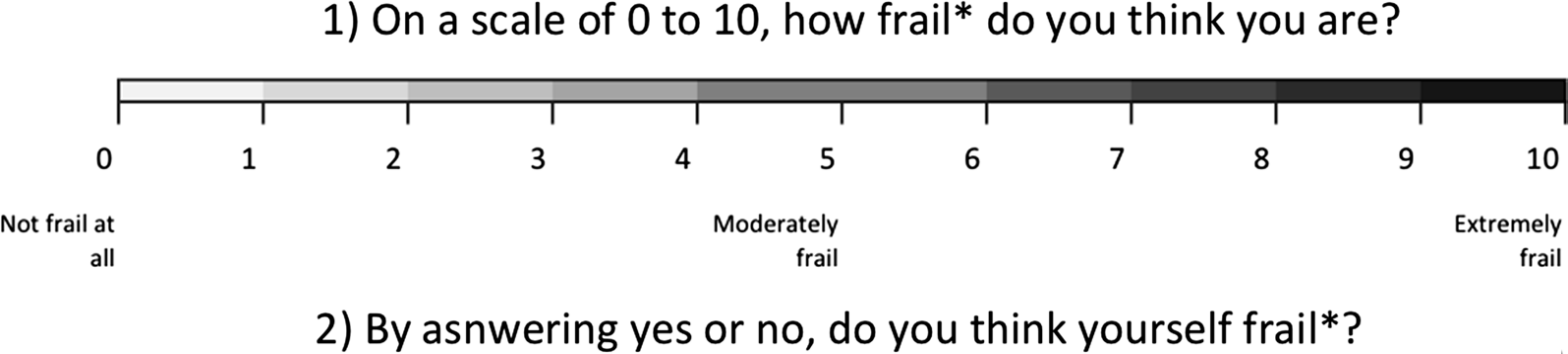
Proposed patient-oriented frailty scales. Asterisk: the definition of frailty needs to be clarified and should be understood by the interviewees

To address the discrepancy between the frailty defined by laypeople and researchers and avoid the problematic assumptions, we developed frailty measures that directly adopted the individual inputs presented in Fig. 1. Before measuring frailty, a definition of frailty should be given and well explained to the participants. The definitions could be adopted from the commonly-used models aforementioned. To determine frailty ranking on a continuous scale, individuals would be asked directly, “on a scale of zero to ten, how frail do you think you are?” In the other question, individuals would be questioned, “by answering yes or no, do you consider yourself to be frail?” to understand whether they consider themselves frail.

Moreover, the new assessment scales were also designed to avoid the problematic assumptions aforementioned and the shortcomings that had been identified in existing frailty indices, listed in Table 2 (a full list published elsewhere [1]). These shortcomings were related to the use of index, the characteristics of the frailty theories, or information collection [1]. Without adopting composite measures for frailty measurement, issues, including the assumptions of equal weights, relatively poorer predictive power than input variables, data processing biases, and excessive complexity of indices [1, 13], could be completely avoided. The theory-based assumptions, such as the thresholds of frailty indices for the diagnosis of frailty status, age criteria, and important components of frailty [13], could be directly assessed with patient perception. The self-rated frailty measures directly introduced patient input and could potentially lead to public engagement by surveying a large population. Also, most frailty indices used existing variables from administrative or survey data [1] and lacked new information that could have the potential to improve our understanding in frailty.

**Table 2:** The issues that a patient-oriented frailty scale might address and those that might emerge

Classifications of the shortcomings	Issues that can be avoided by a patient-oriented frailty scale	Issues merging if patient-oriented frailty scales in use
Index related	1. Unclear rationales for equal weighting of domain variables that leads to unequal weighting of input variables and inclusion of duplicate information	
	2. Biases introduced by data processing that is not based on evidence	
	3. Reproducibility limited by measurement devices and data processing	1. Subjective measurement
	4. Disconnection between frailty theories and produced indices because of excessive numbers of input variables and biases introduced due to data processing	
	5. Complex indices that can be simplified	
	6. Constraints on the regression coefficients of input or domain variables	
	7. Relatively poorer predictive power regarding mortality than input variables	2. Predictive power to be tested
Frailty theory-related	8. Arbitrary thresholds of frailty indices for the diagnosis of frailty statuses	
	9. Arbitrary assumptions about frailty distribution, age correlation, and input variable eligibility of input variables	
	10. Potential disconnection between biology of frailty and the measurement	
	11. Patients’ and the public’ perspectives ignored	3. Deviation from researchers’ definitions
	12. Disconnection to socio-economic determinants	4. Questions on socioeconomic status may deter some to respond
Information generation	13. Old information shuffled, if frailty estimated based on available research or administrative data	5. Reliability and validity to be tested

There were other successful precedents that adopted subjective measures to understand individual health status. One of the most prominent examples was self-rated health status that had been proven important for the prediction of mortality [14], depression [15], cardiovascular disease, diabetes, and other adverse events [16]. This subjective measure was examined and proven valid in the population [17]. Self-rating was also applied in mental health research [18]. For aging research, the subjective measures of frailty and the important components had not been sufficiently studied. Ideally, even the components in the frailty models could be investigated for the usefulness of the subjective measurement. For example, the five items in the Phenotype Model could be measured by subjective judgements. In fact, four of the five items in the Phenotype Model could be measured subjectively with validated questionnaires: weight loss [19], exhaustion [20], walking speed [21], and physical activity [22]. It might be the time to understand the usefulness of subjective frail measures and the relationships between subjective and objective frailty measures.

## Limitations

However, it would be possible that the proposed scales might be subject to certain limitations. There were several potential obstacles for the wide adoption of the subjective measures of frailty. Although there was some consensus on the definition of frailty [12, 23], it remained uncertain whether this consensus would be understood and accepted by most laypeople or researchers. It had been reported that some seniors might not prefer the term, “frail” [24]. Interviewees might be unwilling to consider and rate themselves frail. The acceptability of the term, “frail”, for self-assessment remained unanswered. The proposed subjective measure of frailty, similar to other patient reported outcomes, such as quality of life [25], needed to be tested in different populations to understand it validity and reliability [26]. In general, indices predicted mortality worse than input variables [13]. However, it remained unclear whether this new measure would predict mortality or other outcomes better than existing frailty indices. Currently, no resources were available for us to conduct feasibility test for the proposed frailty measure. Lastly, we did a limited literature search for the definitions of frailty. A systematic search for all frailty definitions might be useful.
